# Susceptibility-Based MRI in Cerebral Arteriovenous Malformations: From Venous Drainage to Physiological Biomarkers—A Narrative Review

**DOI:** 10.3390/biomedicines14051121

**Published:** 2026-05-15

**Authors:** Karol Wiśniewski, Takashi Iimori, Yasuaki Inoue

**Affiliations:** 1 Department of Neurosurgery, Nadogaya Hospital, Chiba 277-0084, Japan; takashi.iimori12@gmail.com (T.I.); inoue.yasuaki@gmail.com (Y.I.); 2 Department of Neurosurgery and Neurooncology, Medical University of Lodz, Kopcinskiego 22, 90-153 Lodz, Poland

**Keywords:** cerebral arteriovenous malformation, susceptibility-weighted imaging, quantitative susceptibility mapping, venous oxygenation, hemodynamic biomarkers

## Abstract

**Background:** Cerebral arteriovenous malformations (AVMs) are high-flow shunts in which abnormal arteriovenous connections expose draining veins to venous hypertension, arterialization, and altered oxygenation. While digital subtraction angiography (DSA) remains the reference standard for dynamic angioarchitecture, it does not directly characterize venous oxygenation or microhemorrhagic tissue changes. **Objective:** To synthesize current evidence on susceptibility-based MRI-susceptibility-weighted imaging (SWI) and quantitative susceptibility mapping (QSM) for characterization, risk-related features, and treatment monitoring in cerebral AVMs. **Methods:** Narrative review of the foundational and contemporary literature on AVM pathophysiology, SWI and QSM technical principles, and clinical applications including venous drainage depiction, microhemorrhage detection, oxygenation-related biomarkers, and post-treatment surveillance. **Results:** SWI provides high-resolution, non-contrast depiction of venous drainage and perinidal hemorrhagic/calcific components, improving visualization of draining veins and microhemorrhages compared with conventional MRI and complementing TOF-MRA. Arterialized draining veins may show altered SWI signal consistent with elevated venous oxygen saturation, though interpretation is indirect and influenced by flow and orientation. QSM extends susceptibility imaging by quantifying tissue susceptibility and enabling indirect estimation of venous oxygenation (SvO_2_), offering a potential physiological biomarker of shunt severity and treatment response after radiosurgery or embolization. Key limitations include lack of dynamic flow timing, flow-related artifacts, orientation dependence, confounding from hemorrhage/calcification, and limited standardization and prospective validation. **Conclusions:** Susceptibility-based MRI does not replace DSA but meaningfully enriches multimodal AVM assessment by adding structural and physiological information-particularly venous mapping, microhemorrhage detection, and oxygenation-sensitive biomarkers. Standardized acquisition/reconstruction and prospective studies are needed to validate susceptibility-derived metrics for risk stratification and longitudinal monitoring.

## 1. Introduction

Cerebral arteriovenous malformations (AVMs) are complex, high-flow vascular lesions characterized by direct arteriovenous shunting through a dysplastic vascular network known as the nidus, without an intervening capillary bed [1]. This fundamental structural abnormality disrupts the physiological pressure gradient normally maintained across the microcirculation. In the absence of capillary resistance, arterial blood is transmitted directly into the venous system, exposing draining veins to elevated flow velocity, increased wall shear stress, and chronic venous hypertension [1,2].

The hemodynamic consequences of this architecture extend beyond simple high flow. Arterialization of draining veins results in profound alterations in venous oxygenation and metabolic exchange. Because blood traverses the nidus rapidly, oxygen extraction may be incomplete, leading to elevated venous oxygen saturation (SvO_2_) within draining veins [3]. This phenomenon has been supported by intraoperative observations of so-called “red veins” and by jugular bulb oxygenation studies demonstrating altered downstream oxygen content [3]. Thus, AVMs are not merely structural anomalies but represent dynamic disturbances in cerebrovascular physiology.

Venous drainage patterns are central to both rupture risk and treatment strategy. Deep venous drainage, venous outflow restriction, and venous stenosis have been repeatedly associated with increased hemorrhagic presentation [4,5,6]. Elevated venous pressure may impair normal cortical venous outflow and reduce cerebrovascular reserve in surrounding brain tissue, contributing to venous congestion and microvascular instability [6]. Histopathological studies have demonstrated degenerative changes within venous walls, including thinning of the media, fragmentation of elastic fibers, and inflammatory remodeling, which may predispose to rupture under chronic hemodynamic stress [7].

In addition to venous hypertension, AVMs may induce regional hypoperfusion in adjacent brain tissue through the “steal phenomenon,” in which high-flow shunting diverts blood away from normal parenchyma [8]. Chronic hypoperfusion can result in impaired autoregulation and altered metabolic demand, further destabilizing the local microenvironment. Molecular studies have also identified increased expression of angiogenic factors such as vascular endothelial growth factor (VEGF), matrix metalloproteinases (MMPs), and inflammatory mediators within AVM tissue, suggesting that these lesions represent biologically active and dynamically remodeling vascular networks rather than static congenital malformations [9,10].

In addition to primary shunt-related hemodynamic alterations, AVMs may exhibit secondary adaptive or pathological vascular remodeling, including the development of fragile collateral networks resembling moyamoya-like vessels. These abnormal, fine, and tortuous collateral channels may arise in response to chronic flow redistribution, regional hypoperfusion, or venous outflow obstruction. Similar to moyamoya disease, these vessels are characterized by structural immaturity, thin walls, and disorganized smooth muscle layers, rendering them vulnerable to rupture. Their presence may reflect chronic hemodynamic stress and compensatory angiogenesis driven by local hypoxia and increased expression of angiogenic mediators such as VEGF [9,10]. Importantly, such fragile collateral networks may not be fully appreciated on conventional angiography unless specifically sought, yet they may contribute to microhemorrhagic events or progressive neurological symptoms. The detection of subtle microvascular remodeling and hemosiderin deposition in these regions further underscores the potential value of susceptibility-based MRI techniques in capturing secondary vascular instability beyond the primary nidus architecture.

The lifetime risk of intracranial hemorrhage in AVM patients remains a major determinant of management strategy. Natural history studies have reported annual hemorrhage rates ranging from 1% to 4%, with higher risk in patients presenting with prior rupture, deep venous drainage, associated aneurysms, or venous outflow abnormalities [4,5]. These clinical observations reinforce the concept that venous hemodynamics—rather than arterial inflow alone—play a pivotal role in AVM instability.

Digital subtraction angiography (DSA) remains the gold standard for delineating AVM angioarchitecture. Its superior temporal resolution allows dynamic visualization of feeding arteries, nidus configuration, early venous filling, and detailed drainage pathways [11]. However, DSA primarily provides anatomical and flow-phase information and does not directly characterize venous oxygenation, microhemorrhagic components, or tissue susceptibility changes. Moreover, it is invasive and associated with procedural risk.

Magnetic resonance imaging (MRI) offers complementary tissue-level assessment. Conventional MRI sequences detect prior hemorrhage, gliosis, and mass effect but may incompletely capture the nuanced physiological alterations within arterialized veins. In this context, susceptibility-based MRI techniques provide a unique opportunity to interrogate both structure and physiology.

Susceptibility-weighted imaging (SWI) exploits magnetic susceptibility differences related to deoxyhemoglobin, hemosiderin, and calcium, enhancing visualization of venous structures and hemorrhagic components [12,13]. Because signal intensity on SWI is strongly influenced by deoxyhemoglobin concentration, arterialized veins with elevated oxygen saturation may demonstrate altered susceptibility characteristics compared with normal cortical veins. Thus, SWI has the potential to reflect underlying hemodynamic states rather than merely depict morphology.

Quantitative susceptibility mapping (QSM) extends this concept by enabling voxel-wise quantification of magnetic susceptibility, which can be used to estimate venous oxygen saturation indirectly [14]. Early investigations have demonstrated elevated SvO_2_ in AVM draining veins, consistent with rapid arteriovenous transit and reduced oxygen extraction [14]. These findings suggest that susceptibility-based imaging may serve as a non-invasive surrogate marker of shunt physiology, venous hypertension, and potentially rupture risk.

Taken together, contemporary understanding of AVMs underscores that these lesions are hemodynamically complex and biologically active entities in which venous pathophysiology plays a central role. Imaging approaches capable of characterizing venous oxygenation, microhemorrhage, and susceptibility-related changes may therefore provide clinically meaningful information beyond conventional angiographic anatomy.

The aim of this narrative review is to synthesize current knowledge regarding susceptibility-based MRI in cerebral AVMs, integrating pathophysiological mechanisms with technical principles and clinical applications. Rather than performing a formal meta-analysis, we seek to contextualize SWI and QSM within the broader framework of AVM biology, rupture risk, and multimodal imaging strategies. This review aims not only to summarize existing evidence but also to provide a pathophysiology-driven framework linking susceptibility-based imaging with AVM hemodynamics and clinical decision-making.

## 2. Search Strategy

A non-systematic literature search was conducted to identify relevant studies on susceptibility-based MRI techniques, including susceptibility-weighted imaging (SWI) and quantitative susceptibility mapping (QSM), in cerebral arteriovenous malformations (AVMs). Electronic databases including PubMed, Scopus, and Web of Science were searched for articles published in English from January 2004 (corresponding to the introduction of susceptibility-weighted imaging into clinical practice) to January 2026.

The search strategy combined keywords and Boolean operators as follows: (“arteriovenous malformation” OR “AVM”) AND (“susceptibility-weighted imaging” OR “SWI” OR “quantitative susceptibility mapping” OR “QSM” OR “susceptibility MRI”). Additional terms related to venous oxygenation, microhemorrhage, and cerebral hemodynamics were also considered.

The combined search across the three databases yielded 20,694 records. Reference lists of relevant articles and review papers were manually screened to identify additional studies not captured in the database search.

**Inclusion criteria** included original research articles and review papers focusing on susceptibility-based MRI techniques (SWI and/or QSM) in cerebral arteriovenous malformations, including studies addressing venous drainage, oxygenation, microhemorrhage detection, hemodynamics, treatment monitoring, or technical aspects of susceptibility imaging.

**Exclusion criteria** included case reports, conference abstracts, editorials, non-English publications, animal studies not directly translatable to cerebral AVM imaging, and studies not specifically related to susceptibility-based MRI in AVMs.

Studies were selected based on their relevance to AVM pathophysiology, imaging characteristics, technical aspects of susceptibility-based MRI, and clinical applications including diagnosis, risk assessment, and treatment monitoring. Both original research articles and review papers were included, while case reports, non-English publications, and studies not directly related to susceptibility imaging in AVMs were excluded.

This approach is consistent with a narrative review methodology and does not follow a formal systematic review protocol.

Representative studies were selected for structured comparison based on their relevance and contribution to key imaging and pathophysiological concepts discussed in this review.

## 3. Technical Background of Susceptibility-Based MRI

### 3.1. Physics of Magnetic Susceptibility

Magnetic susceptibility describes the degree to which a material becomes magnetized in an external magnetic field. In biological tissues, susceptibility differences arise primarily from variations in iron content, blood oxygenation, calcium deposition, and other paramagnetic or diamagnetic substances [15]. These susceptibility variations affect signal intensity and phase on gradient-echo MRI.

A central mechanism underlying susceptibility-based imaging is the oxygenation-dependent magnetic behavior of hemoglobin. Oxygenated hemoglobin is diamagnetic, whereas deoxygenated hemoglobin is paramagnetic. The presence of paramagnetic deoxyhemoglobin within venous blood produces local magnetic field distortions, leading to T2* shortening and phase shifts in gradient-echo acquisitions [16,17]. As a result, venous structures containing higher concentrations of deoxyhemoglobin appear hypointense on susceptibility-sensitive magnitude images.

This oxygenation-dependent susceptibility effect forms the basis of the blood oxygen level-dependent (BOLD) phenomenon [16]. Originally described in functional MRI, the BOLD signal arises from changes in deoxyhemoglobin concentration within the microvasculature, altering local magnetic susceptibility and therefore signal intensity [16,18]. In the context of cerebral AVMs, where rapid arteriovenous transit may reduce oxygen extraction, susceptibility characteristics of draining veins may differ from those of normal cortical veins, reflecting altered shunt physiology.

In addition to hemoglobin, susceptibility-based imaging differentiates paramagnetic substances such as hemosiderin and ferritin from diamagnetic materials such as calcium [19]. This distinction is particularly relevant in AVMs, where chronic microhemorrhage, prior rupture, or calcified nidus components may coexist.

### 3.2. Susceptibility-Weighted Imaging (SWI)

Susceptibility-weighted imaging (SWI) is a high-resolution, three-dimensional, fully flow-compensated gradient-echo technique that combines magnitude and filtered phase information to enhance susceptibility contrast [20]. Unlike conventional T2*-weighted imaging, which relies primarily on magnitude signal loss due to dephasing, SWI explicitly integrates phase data to amplify differences between tissues with distinct magnetic properties [20,21].

SWI combines magnitude and filtered phase information to enhance venous and hemorrhagic contrast, often using minimum intensity projection (minIP) reconstructions to improve venous conspicuity.

Magnitude images often provide superior delineation of the nidus and draining veins compared with conventional sequences [22]. The signal behavior of draining veins on SWI reflects a combination of deoxyhemoglobin concentration, inflow effects, and flow velocity. Normal cortical veins typically appear hypointense due to higher deoxyhemoglobin content. However, arterialized draining veins in AVMs may demonstrate relative hyperintensity or mixed signal patterns on magnitude images, possibly reflecting elevated oxygen saturation and altered susceptibility gradients [23].

Minimum intensity projection (minIP) enhances continuity of venous structures and increases sensitivity to microhemorrhage but may reduce fine spatial detail due to projection averaging [20]. Phase images are particularly useful for differentiating paramagnetic blood products (negative phase shift) from diamagnetic calcium (positive phase shift), allowing more accurate characterization of complex nidus composition [19,24].

Compared with conventional T2*-weighted gradient-echo imaging, SWI provides:

- Higher spatial resolution;
- Increased sensitivity to microhemorrhage;
- Improved venous conspicuity;
- Enhanced differentiation between calcification and hemorrhage.

However, SWI remains qualitative and is influenced by vessel orientation, field strength, and local susceptibility gradients.

### 3.3. Quantitative Susceptibility Mapping (QSM)

Quantitative susceptibility mapping (QSM) is an advanced reconstruction technique that estimates tissue magnetic susceptibility values from phase data acquired during gradient-echo imaging [25]. Unlike SWI, which provides qualitative susceptibility contrast, QSM enables voxel-wise quantitative assessment of tissue susceptibility [25,26].

QSM reconstruction involves phase processing, background field removal, and dipole inversion to generate quantitative susceptibility maps.

Through this process, QSM generates quantitative maps reflecting tissue magnetic properties, which can be related to deoxyhemoglobin concentration and, indirectly, venous oxygen saturation (SvO_2_) [26,27]. In cerebral AVMs, QSM studies have demonstrated elevated susceptibility-derived oxygenation values in draining veins, consistent with incomplete oxygen extraction due to rapid arteriovenous shunting [14].

By enabling non-invasive estimation of SvO_2_, QSM extends susceptibility imaging beyond structural venography toward functional hemodynamic assessment. This capability is particularly relevant in AVMs, where venous oxygenation may reflect shunt severity and altered metabolic exchange.

### 3.4. Technical Limitations

Key technical limitations include:

- **Flow-related artifacts**: Rapid blood flow within the nidus or draining veins may produce phase dispersion and signal loss, compromising susceptibility estimation [28].
- **Orientation dependence**: Susceptibility measurements vary with vessel orientation relative to the main magnetic field (B_0_), potentially affecting quantitative accuracy [29].
- **Hemosiderin contamination**: Prior hemorrhage introduces strong paramagnetic signals that may confound interpretation of venous oxygenation [19].
- **Partial volume effects**: Small vessels may not be adequately resolved, especially at 1.5 T field strength.
- **Algorithmic variability**: Differences in phase processing and dipole inversion methods limit inter-study reproducibility [26].

Therefore, although QSM provides physiologically meaningful information, interpretation in AVMs requires correlation with angiographic and structural imaging findings. Recent methodological developments have focused on improving QSM reconstruction stability and inter-site reproducibility, highlighting the importance of standardized processing pipelines.

## 4. Visualization of AVM Angioarchitecture on SWI

Susceptibility-weighted imaging (SWI) provides unique structural information regarding the venous and hemorrhagic components of cerebral arteriovenous malformations. While SWI does not replace digital subtraction angiography (DSA) for dynamic vascular mapping, it enhances morphological delineation of specific angioarchitectural features, particularly the nidus and venous drainage system.

### 4.1. Nidus Delineation

The nidus of an AVM consists of a compact network of dysplastic, tortuous vessels with variable flow velocities and heterogeneous blood oxygenation. On conventional MRI sequences, the nidus may appear as a flow void or mixed-signal vascular conglomerate; however, spatial resolution and contrast may be insufficient to clearly distinguish its internal architecture from adjacent hemorrhage or calcification.

On SWI magnitude images, the nidus often demonstrates improved conspicuity due to susceptibility contrast generated by intranidal blood products, microhemorrhage, and variable deoxyhemoglobin content [12]. The integration of magnitude and phase data enhances differentiation between paramagnetic hemorrhagic components and diamagnetic calcifications, which may coexist within the nidus [19]. This capability is particularly relevant in partially thrombosed or chronically hemorrhagic AVMs, where internal composition may influence both rupture risk and surgical strategy.

The improved contrast resolution of SWI magnitude images can assist in defining the spatial boundaries of the nidus relative to surrounding brain parenchyma. In microsurgical planning, accurate nidus delineation is critical for identifying margins, avoiding premature venous occlusion, and preserving eloquent cortex. Although DSA remains essential for assessing feeding arteries and dynamic flow, SWI may provide complementary information regarding parenchymal infiltration, hemosiderin deposition, and adjacent venous congestion [13].

Furthermore, detection of microhemorrhagic foci surrounding the nidus may suggest prior subclinical rupture, which has implications for risk stratification and surgical urgency [30]. Thus, SWI contributes not only to structural visualization but also to biological characterization of the lesion.

### 4.2. Draining Veins

The venous drainage system is a central determinant of AVM instability and hemorrhagic risk. SWI is particularly sensitive to venous structures due to its susceptibility-based contrast mechanism.

### 4.3. Mechanism of Hyperintensity in Arterialized Veins

Under normal physiological conditions, cortical veins contain higher concentrations of deoxyhemoglobin and therefore appear hypointense on susceptibility-sensitive sequences. In AVMs, however, rapid arteriovenous transit and incomplete oxygen extraction may lead to elevated venous oxygen saturation (SvO_2_), reducing local susceptibility gradients [3]. This phenomenon can result in relative hyperintensity or mixed signal intensity of arterialized draining veins on SWI magnitude images [23].

In addition to oxygenation changes, inflow effects and high flow velocity within draining veins may contribute to altered signal characteristics. Fully flow-compensated gradient-echo sequences used in SWI can partially preserve signal from rapidly flowing blood, further influencing venous appearance [31]. Consequently, arterialized veins may demonstrate signal patterns distinct from normal cortical veins, potentially serving as indirect imaging markers of shunt physiology.

### 4.4. Comparison with TOF-MRA

Time-of-flight magnetic resonance angiography (TOF-MRA) relies on inflow enhancement to visualize high-velocity arterial blood but is less sensitive to venous structures, particularly slow-flow or small-caliber veins [32]. While TOF-MRA excels in delineating feeding arteries, it may incompletely depict complex venous drainage patterns.

Several studies have demonstrated that SWI detects a higher number of draining veins compared with conventional MR angiography techniques [23,33]. Phase-filtered SWI images may enhance small venous channels that are not readily visible on TOF-MRA. However, TOF-MRA may outperform SWI in identifying very high-flow components due to strong inflow enhancement [23]. Thus, SWI and TOF-MRA should be viewed as complementary rather than competing techniques.

### 4.5. Assessment of Superficial vs. Deep Drainage

Differentiation between superficial cortical drainage and deep venous drainage is clinically critical, as deep drainage is incorporated into grading systems and associated with higher hemorrhage risk [4,5]. SWI can aid in visualizing venous trajectories toward deep structures such as the internal cerebral veins, basal veins, or straight sinus.

Because SWI enhances venous conspicuity without reliance on contrast injection, it allows detailed mapping of venous pathways in a non-invasive manner. In selected cases, SWI may clarify whether venous outflow involves exclusively superficial cortical veins or extends to deep venous structures [33]. However, precise confirmation of venous phase timing and dynamic flow characteristics still requires DSA.

### 4.6. Feeding Arteries: SWI Limitations

While SWI is highly sensitive to venous and hemorrhagic components, it is inherently limited in evaluating feeding arteries. Several factors contribute to this limitation:

1. **Reduced susceptibility contrast in oxygenated arterial blood**
  Arterial blood is predominantly oxygenated and therefore diamagnetic, generating minimal susceptibility-induced signal loss [15,16]. As a result, feeding arteries may not demonstrate prominent contrast relative to surrounding tissue on SWI.
2. **Flow-related signal behavior**
  Although SWI sequences are flow-compensated, very high flow velocities within feeding arteries may lead to signal loss or inconsistent visualization depending on acquisition parameters [31].
3. **Lack of dynamic information**
  SWI provides static structural images and does not capture temporal information regarding arterial inflow, early venous filling, or shunt timing. Dynamic characterization of feeder hierarchy and flow direction remains the domain of DSA [11].
4. **Orientation dependence and partial volume effects**
  Small feeding arteries, particularly when oriented parallel to the main magnetic field (B_0_), may generate minimal susceptibility contrast and be partially obscured [29].

Therefore, while SWI enhances visualization of venous drainage and microhemorrhage, it does not reliably replace angiographic techniques for detailed analysis of arterial feeders. Multimodal imaging remains essential, with SWI providing structural and physiological context and DSA supplying dynamic vascular mapping. These limitations are discussed in more detail in Section 8.

## 5. Hemodynamic and Physiologic Insight

Beyond structural delineation of angioarchitecture, susceptibility-based MRI may provide insight into selected physiological consequences of arteriovenous shunting, particularly venous oxygenation and microhemorrhagic instability. These mechanisms have been introduced above; this section focuses on their imaging correlates and potential clinical relevance.

### 5.1. Venous Oxygen Saturation (QSM and Estimation of SvO_2_)

Quantitative susceptibility mapping (QSM) enables voxel-wise estimation of magnetic susceptibility values, which can be related to deoxyhemoglobin concentration and, indirectly, venous oxygen saturation (SvO_2_) [25,26]. In venous blood, susceptibility differences arise primarily from the paramagnetic properties of deoxyhemoglobin. By modeling these differences, QSM allows indirect calculation of oxygen extraction and relative venous oxygenation.

In cerebral AVMs, elevated SvO_2_ within draining veins may reflect rapid arteriovenous transit and incomplete oxygen extraction [14]. QSM studies have demonstrated increased susceptibility-derived oxygenation values in AVM draining veins compared with normal cortical veins, supporting the concept of venous arterialization [14]. These findings align with classical hemodynamic observations of “red veins” seen intraoperatively in AVM surgery [3].

Unlike DSA, which visualizes dynamic flow but does not directly quantify oxygenation, QSM offers a non-invasive surrogate marker of shunt physiology. Elevated venous oxygen saturation may reflect high-flow, low-resistance shunting, whereas normalization of SvO_2_ following radiosurgery or embolization could indicate successful hemodynamic modulation [14]. Thus, QSM has potential utility not only in baseline evaluation but also in longitudinal monitoring.

However, it should be emphasized that the use of QSM-derived parameters as clinical biomarkers remains exploratory. Current evidence is limited, and these measures have not yet been prospectively validated for predicting rupture risk or guiding treatment decisions.

### 5.2. “Red Vein” as an Imaging Correlate

The term “red vein” historically refers to the bright-red appearance of draining veins during surgical exposure of AVMs, reflecting higher oxygen content relative to normal venous blood [3]. On susceptibility-sensitive MRI, this phenomenon may manifest as altered signal intensity in arterialized veins.

Because normal veins appear hypointense on SWI due to higher deoxyhemoglobin concentration, arterialized veins with elevated SvO_2_ may demonstrate relative hyperintensity or reduced susceptibility-related signal loss [23,34]. While signal intensity on SWI is influenced by multiple factors—including flow velocity and vessel orientation—the combination of magnitude and phase characteristics may provide indirect evidence of altered venous oxygenation.

### 5.3. Venous Congestion and Hemorrhagic Risk

Venous outflow impairment and congestion are central mechanisms in AVM instability and rupture, as outlined in the Introduction. In this context, susceptibility-based MRI may help identify indirect imaging markers of venous stress [6,7].

### 5.4. Hypointensity vs. Hyperintensity on SWI

Signal characteristics of draining veins on SWI may reflect underlying hemodynamic states. Hypointense veins typically contain higher concentrations of deoxyhemoglobin, consistent with normal oxygen extraction or venous stasis. In contrast, relative hyperintensity may suggest arterialization and reduced susceptibility gradients [23].

Interestingly, some studies have suggested that marked hypointensity in draining veins may indicate preserved venous flow and lower hemorrhagic risk, whereas altered or heterogeneous signal patterns could reflect congestion or abnormal hemodynamics [34]. The relationship between susceptibility signal patterns and rupture risk remains incompletely defined; however, susceptibility imaging may reveal subtle alterations not apparent on angiography.

### 5.5. Potential Imaging Biomarkers of ICH Risk

Beyond gross angioarchitectural features, susceptibility-based imaging may identify microstructural markers associated with hemorrhagic instability. These include:

- Focal microhemorrhages adjacent to draining veins;
- Perinidal hemosiderin deposition;
- Venous wall irregularity;
- Susceptibility halos indicating chronic leakage.

Microbleeds detected on SWI may represent prior subclinical rupture events and have been associated with increased risk of subsequent hemorrhage in other cerebrovascular diseases [30]. In AVMs, detection of occult microhemorrhage may identify biologically active lesions at higher rupture risk, even in the absence of overt clinical hemorrhage.

While prospective validation is lacking, the integration of susceptibility-derived markers with established risk factors (deep drainage, associated aneurysms, prior hemorrhage) may enhance individualized risk assessment.

### 5.6. Microhemorrhage and Hemosiderin Detection

One of the most established strengths of SWI is its sensitivity to microhemorrhage and iron deposition. Hemosiderin, a paramagnetic blood breakdown product, produces strong local susceptibility effects and appears as marked hypointensity on magnitude images [19,30].

In AVMs, chronic low-grade leakage from fragile venous structures or moyamoya-like collateral networks may result in perinidal hemosiderin deposition. These deposits may be inapparent on conventional T2-weighted imaging but conspicuous on SWI [12]. The detection of microhemorrhages surrounding the nidus or along draining veins may indicate repeated minor bleeding episodes and progressive vascular instability.

Importantly, differentiation between calcification and hemorrhage is facilitated by phase imaging, as diamagnetic calcium produces phase shifts opposite to those of paramagnetic blood products [19]. This distinction is clinically relevant in AVMs with partially calcified nidus components.

Beyond diagnostic utility, the presence of microhemorrhages may influence management decisions. Detection of occult hemorrhagic activity may prompt closer surveillance, earlier intervention, or reconsideration of treatment modality. In radiosurgical follow-up, persistence or emergence of new susceptibility foci could suggest ongoing hemodynamic stress or incomplete obliteration.

Thus, susceptibility-based MRI extends the evaluation of AVMs from static angioarchitecture toward dynamic biological characterization, incorporating venous oxygenation, congestion, and microhemorrhagic activity into a unified imaging framework.

However, reported findings across studies are not fully consistent, particularly regarding the interpretation of susceptibility signal changes and their relationship to venous oxygenation and hemorrhage risk. Differences in imaging protocols, field strength, and reconstruction methods contribute to variability in results and limit direct comparability between studies.

## 6. SWI in Treatment Planning and Follow-Up

Susceptibility-weighted imaging (SWI) and quantitative susceptibility mapping (QSM) provide structural and physiological information that may complement angiographic assessment during treatment planning and longitudinal follow-up of cerebral arteriovenous malformations (AVMs). Although digital subtraction angiography (DSA) remains indispensable for dynamic vascular mapping, susceptibility-based imaging contributes additional insight into nidus characterization, venous hemodynamics, and hemorrhagic evolution. The limitations of susceptibility-based imaging are discussed in detail in Section 8.

### 6.1. Preoperative Assessment—Nidus Delineation

Accurate delineation of the nidus is essential for microsurgical planning, radiosurgical targeting, and endovascular strategy. While DSA defines arterial feeders and venous drainage timing, it may incompletely characterize the spatial relationship between the nidus and adjacent parenchyma. SWI magnitude images enhance contrast between vascular structures, hemorrhagic components, and surrounding brain tissue, improving delineation of nidus margins [12,21].

In partially thrombosed or chronically hemorrhagic AVMs, SWI is particularly valuable for identifying intranidal hemosiderin deposition and differentiating hemorrhage from calcification using phase information [19]. This distinction may influence operative planning, as calcified segments may exhibit altered mechanical properties and embolization response.

Detection of perinidal microhemorrhages may suggest biologically active regions within the malformation [30]. Recognition of such features can inform surgical prioritization and risk discussion.

### 6.2. Preoperative Assessment—Evaluation of Venous Drainage

Preservation of draining veins until complete nidus disconnection is a cornerstone of microsurgical AVM resection. Premature venous occlusion may precipitate catastrophic hemorrhage. SWI enhances visualization of superficial and deep venous pathways without the need for contrast administration [23,33].

In selected cases, SWI may clarify whether venous drainage is exclusively cortical or involves deep venous structures, which is critical for grading and surgical risk stratification [4]. Although DSA remains required for dynamic assessment, susceptibility imaging may provide additional anatomical context in preoperative mapping.

Integration of SWI into neuronavigation platforms may further assist in identifying venous corridors intraoperatively, particularly in eloquent or deep-seated lesions.

### 6.3. Post-Radiosurgery Evaluation

Stereotactic radiosurgery induces progressive endothelial proliferation, intimal thickening, and gradual obliteration of the nidus over months to years [35]. During this latency period, patients remain at risk of hemorrhage, making imaging surveillance essential.

### 6.4. Changes in Venous Oxygen Saturation

QSM offers a potential method for monitoring hemodynamic changes following radiosurgery. As the shunt progressively closes, arteriovenous transit time may normalize, leading to increased oxygen extraction and reduced venous oxygen saturation [14]. This physiological shift may manifest as increased susceptibility-related hypointensity in draining veins over time.

Preliminary studies suggest that normalization of venous susceptibility characteristics may correlate with progressive shunt reduction [14]. Although these findings require further validation, QSM could theoretically provide early non-invasive indicators of treatment response before angiographic obliteration is complete.

### 6.5. Obliteration vs. Persistent Shunt

Complete obliteration is typically confirmed by DSA; however, susceptibility-based imaging may provide supportive evidence. Persistent hyperintense or mixed-signal draining veins on SWI may indicate ongoing arterialization and residual shunting [23,34]. Conversely, disappearance or normalization of abnormal venous signal patterns may suggest hemodynamic stabilization.

In addition, SWI is highly sensitive to radiation-induced microhemorrhage or cavernoma-like changes within treated regions [36]. Differentiating post-treatment hemorrhagic sequelae from residual nidus components is critical in long-term follow-up.

Thus, susceptibility imaging complements angiographic evaluation by providing tissue-level and physiological information during the radiosurgical latency period.

### 6.6. Monitoring After Embolization

Endovascular embolization is frequently employed as a standalone therapy or adjunct to surgery or radiosurgery. Embolization materials such as Onyx or n-butyl cyanoacrylate alter intranidal flow dynamics and may introduce susceptibility artifacts.

### 6.7. Hemodynamic Changes

Successful embolization reduces shunt flow and modifies venous hemodynamics. In theory, reduced arteriovenous transit may increase oxygen extraction and normalize venous oxygen saturation. QSM could potentially detect these changes through shifts in susceptibility-derived SvO_2_ estimates [14].

SWI may also reveal decreased prominence of arterialized draining veins following effective embolization. However, interpretation must consider susceptibility artifacts introduced by embolic agents, which can produce signal voids or phase distortion [32].

### 6.8. Detection of Residual Nidus

Residual nidus after embolization may be difficult to identify on conventional MRI. SWI may assist by highlighting persistent abnormal venous drainage or residual microhemorrhagic activity [12]. Persistent arterialized venous signal patterns could suggest incomplete shunt occlusion.

### 6.9. Post-Embolization Complications

SWI is particularly sensitive to procedure-related microhemorrhage or venous thrombosis. Early detection of hemorrhagic complications may alter clinical management and surveillance intervals [30]. Additionally, evaluation of venous outflow patency is essential, as inadvertent venous occlusion may precipitate delayed hemorrhage.

Collectively, susceptibility-based imaging expands the role of MRI in AVM management from diagnostic adjunct to longitudinal physiological monitoring tool. While DSA remains indispensable for definitive assessment of obliteration, SWI and QSM provide complementary structural and hemodynamic insights that may refine treatment planning and follow-up strategies.

## 7. Comparison—Imaging Modalities for AVMs

The evaluation of cerebral arteriovenous malformations requires a multimodal imaging strategy that integrates dynamic vascular mapping with high-resolution structural and physiological assessment. Each imaging modality provides distinct advantages and limitations, and susceptibility-based techniques occupy a complementary role within this framework (Table 1). A structured summary of representative studies is presented in Table 2.

### 7.1. Digital Subtraction Angiography (DSA)

DSA remains the gold standard for AVM evaluation due to its unmatched spatial and temporal resolution [37]. It provides dynamic visualization of arterial inflow, nidus architecture, early venous filling, and drainage timing, allowing precise characterization of shunt physiology. DSA is indispensable for treatment planning and confirmation of complete obliteration.

However, DSA is invasive and associated with procedural risks including stroke, arterial injury, and contrast-related complications [38]. Moreover, while DSA excels in flow-phase visualization, it does not provide direct information about venous oxygenation, microhemorrhage, or tissue susceptibility characteristics.

### 7.2. Time-of-Flight Magnetic Resonance Angiography (TOF-MRA)

TOF-MRA relies on inflow enhancement of unsaturated spins to depict high-velocity arterial blood [32]. It is particularly effective in identifying feeding arteries and large-caliber vessels. However, TOF-MRA is less sensitive to venous structures, especially those with slower or complex flow patterns.

Temporal resolution remains limited compared to DSA, and differentiation between arterial and venous phases may be challenging in high-flow AVMs [32]. While non-invasive, TOF-MRA provides primarily morphological information without direct physiological insight into oxygenation or venous congestion.

### 7.3. Susceptibility-Weighted Imaging (SWI)

SWI offers better detection of venous structures and microhemorrhage compared with conventional MRI sequences [20,21]. It enhances depiction of draining veins, perinidal hemosiderin deposition, and calcification. Unlike TOF-MRA, SWI does not rely on inflow enhancement but instead exploits intrinsic magnetic susceptibility differences.

Hemodynamic interpretation remains indirect and dependent on signal characteristics influenced by multiple factors, including flow velocity and oxygenation [23].

### 7.4. Quantitative Susceptibility Mapping (QSM)

QSM extends susceptibility imaging into the quantitative domain by estimating tissue magnetic susceptibility and indirectly assessing venous oxygen saturation [25,26]. In AVMs, QSM offers potential insight into shunt physiology and treatment response.

Nevertheless, QSM does not provide real-time flow dynamics and remains technically demanding. Its role is currently investigational and complementary rather than substitutive for angiography.

Taken together, susceptibility-based imaging does not replace DSA but enriches the diagnostic and monitoring framework by adding structural and physiological dimensions that are not captured by conventional angiography.

Table 2 summarizes representative studies selected based on their direct relevance to susceptibility-based imaging in AVMs. The number of included studies was intentionally limited due to the relatively small body of available literature, particularly for QSM, and substantial heterogeneity in study design, imaging protocols, and outcome measures, which limits direct comparability.

## 8. Limitations of Susceptibility-Based Imaging

Despite its promise, susceptibility-based imaging in AVMs has several important limitations that must be acknowledged.

### 8.1. Lack of Dynamic Flow Assessment

SWI and QSM provide static structural and susceptibility-based information but do not capture dynamic arterial inflow, shunt timing, or venous phase transitions. Dynamic flow assessment remains the domain of DSA and, to a lesser extent, time-resolved contrast-enhanced MRA [37,39]. Consequently, susceptibility-based techniques cannot independently characterize feeder hierarchy or precise arteriovenous transit timing.

### 8.2. Flow-Related Artifacts

High-velocity or turbulent flow within the nidus and draining veins may introduce phase dispersion and signal loss, potentially confounding interpretation [28]. Rapid flow may produce inconsistent susceptibility measurements or obscure small-caliber vessels. Moreover, embolization materials such as Onyx may generate susceptibility artifacts that limit evaluation of treated regions [40].

### 8.3. Orientation Dependence

Susceptibility contrast is influenced by vessel orientation relative to the main magnetic field (B_0_). Vessels aligned parallel to B_0_ may demonstrate reduced susceptibility effects compared with those oriented perpendicularly [29]. This anisotropy may introduce variability in quantitative susceptibility estimation and complicate cross-patient comparisons.

### 8.4. Limited Standardization of QSM

QSM reconstruction requires complex post-processing steps, including phase unwrapping and dipole inversion. Variability in acquisition parameters, filtering techniques, and inversion algorithms may lead to inconsistent susceptibility measurements across centers [41,42]. Lack of standardized protocols limits reproducibility and widespread clinical adoption. Recent studies have emphasized the need for harmonized acquisition and reconstruction pipelines, as variability in phase processing, background field removal, and dipole inversion algorithms may significantly affect quantitative susceptibility values across centers [43,44].

### 8.5. Influence of Hemosiderin and Calcification

Strong paramagnetic effects from prior hemorrhage may confound the interpretation of venous oxygenation on QSM. Similarly, differentiation between calcifications and blood products requires careful phase analysis [19]. In AVMs with complex internal composition, susceptibility signals may reflect multiple overlapping sources. Further research on AVM rupture risk factors is needed to develop more accurate risk stratification scales [45]; therefore, SWI may play a useful role in this context.

### 8.6. Limited Prospective Validation

Although susceptibility-based imaging has demonstrated promising correlations with venous physiology and microhemorrhage detection, robust prospective validation of susceptibility-derived metrics remains limited. In particular, quantitative susceptibility mapping (QSM)-based estimation of venous oxygen saturation (SvO_2_) and susceptibility-weighted imaging (SWI)-based detection and quantification of microhemorrhages have not yet been validated as reliable predictors of rupture risk or treatment response in prospective cohorts.

Current evidence is largely derived from retrospective, single-center studies with limited sample sizes, precluding the establishment of clinically actionable thresholds. For these parameters to be incorporated into routine clinical decision-making, large prospective, multicenter studies are required, with standardized acquisition protocols, reproducible reconstruction pipelines, and predefined quantitative thresholds (e.g., SvO_2_ ranges or microhemorrhage burden) correlated with clinical endpoints such as hemorrhage occurrence or treatment outcomes.

Until such validation is achieved, susceptibility-derived metrics should be considered exploratory and interpreted within a multimodal imaging framework rather than as standalone predictors.

Accordingly, QSM-based biomarkers such as SvO_2_ estimation should currently be interpreted with caution and within a research context.

Overall, the strength of evidence remains limited, as most available data are derived from retrospective studies with small cohorts and heterogeneous methodologies.

### 8.7. Narrative Review Design and Heterogeneity of Available Studies

A formal meta-analysis was not performed because the available literature is highly heterogeneous. Studies differ substantially in MRI acquisition parameters (including field strength and SWI protocols), QSM reconstruction methods, outcome measures, and patient populations. In addition, most published studies are retrospective and involve relatively small cohorts. These methodological differences limit direct comparability between studies and preclude meaningful pooling of effect sizes.

## 9. Future Directions

The future development of susceptibility-based MRI in cerebral arteriovenous malformations (AVMs) lies in multimodal integration, ultra-high-field imaging, advanced computational analysis, and biomarker validation.

A key direction is the integration of susceptibility-weighted imaging (SWI), arterial spin labeling (ASL), and quantitative susceptibility mapping (QSM) into unified acquisition protocols. While SWI provides detailed visualization of venous drainage and microhemorrhage, ASL enables non-invasive assessment of perfusion and steal phenomena, and QSM offers indirect quantification of venous oxygen saturation. Combined analysis of these complementary datasets may allow simultaneous characterization of angioarchitecture, regional perfusion, and shunt physiology within a single examination. Such multiparametric approaches could improve biological risk stratification beyond morphology alone.

Ultra-high-field imaging at 7 Tesla represents another promising avenue. Increased signal-to-noise ratio and enhanced susceptibility contrast may improve detection of small draining veins, subtle microhemorrhages, and intranidal structural heterogeneity. However, technical challenges including field inhomogeneity, artifact susceptibility, and limited availability must be addressed before widespread clinical adoption. Machine learning-based image analysis may further enhance reproducibility and quantitative assessment. Recent advances in deep learning have introduced novel approaches for QSM reconstruction and artifact correction, improving robustness to noise, reducing streaking artifacts, and accelerating reconstruction pipelines [46,47]. These developments may facilitate broader clinical implementation of susceptibility-based imaging. Automated venous segmentation, extraction of morphometric features, and classification of arterialized venous signal patterns could reduce interobserver variability and generate objective imaging metrics.

Ultimately, prospective longitudinal studies are required to validate susceptibility-derived parameters as imaging biomarkers of hemorrhage risk and treatment response. Standardized acquisition protocols and structured reporting frameworks will be essential to ensure reproducibility and facilitate integration of susceptibility-based MRI into routine AVM evaluation.

## 10. Proposed Clinical Integration Model

A pragmatic clinical integration model for susceptibility-based MRI in cerebral arteriovenous malformations (AVMs) should preserve the central role of digital subtraction angiography (DSA) while incorporating susceptibility techniques as complementary tools within a stepwise diagnostic and monitoring algorithm.

***Step 1*** *. **Baseline MRI Including SWI***

Initial evaluation should include standard brain MRI sequences (T1-weighted, T2-weighted, FLAIR, and T2*-weighted imaging) combined with susceptibility-weighted imaging (SWI). This approach enables detection of prior hemorrhage, microhemorrhages, perinidal hemosiderin deposition, venous congestion, and abnormal venous signal patterns suggestive of arterialization. SWI enhances visualization of draining veins and may identify occult microvascular instability not apparent on conventional sequences. In patients with incidentally detected vascular lesions or unexplained hemorrhage, this combined MRI protocol serves as a comprehensive non-invasive screening step.

***Step 2*** *. **Angiographic Confirmation with DSA in Suspected Shunt***

If MRI findings suggest an arteriovenous shunt—such as enlarged draining veins, abnormal susceptibility patterns, or flow voids—DSA should be performed for definitive characterization. DSA remains indispensable for detailed evaluation of feeding arteries, nidus architecture, venous drainage timing, and treatment planning. Susceptibility findings may guide targeted angiographic assessment but do not replace dynamic vascular mapping.

***Step 3*** *. **QSM as a Research and Prognostic Tool***

Quantitative susceptibility mapping (QSM) should currently be regarded as an adjunctive, investigational modality. However, it should be emphasized that susceptibility-derived metrics, including SvO_2_ estimation and microhemorrhage burden, currently lack prospective validation for predicting rupture risk or guiding treatment decisions. Their integration into clinical practice will require well-designed prospective studies with clearly defined quantitative thresholds and outcome-based validation.

It may provide indirect estimation of venous oxygen saturation and offer insight into shunt physiology, treatment response, and potential hemorrhage risk. As standardization improves and prospective validation emerges, QSM-derived parameters may evolve into clinically actionable biomarkers integrated into longitudinal follow-up and risk stratification models.

**Figure 1:**
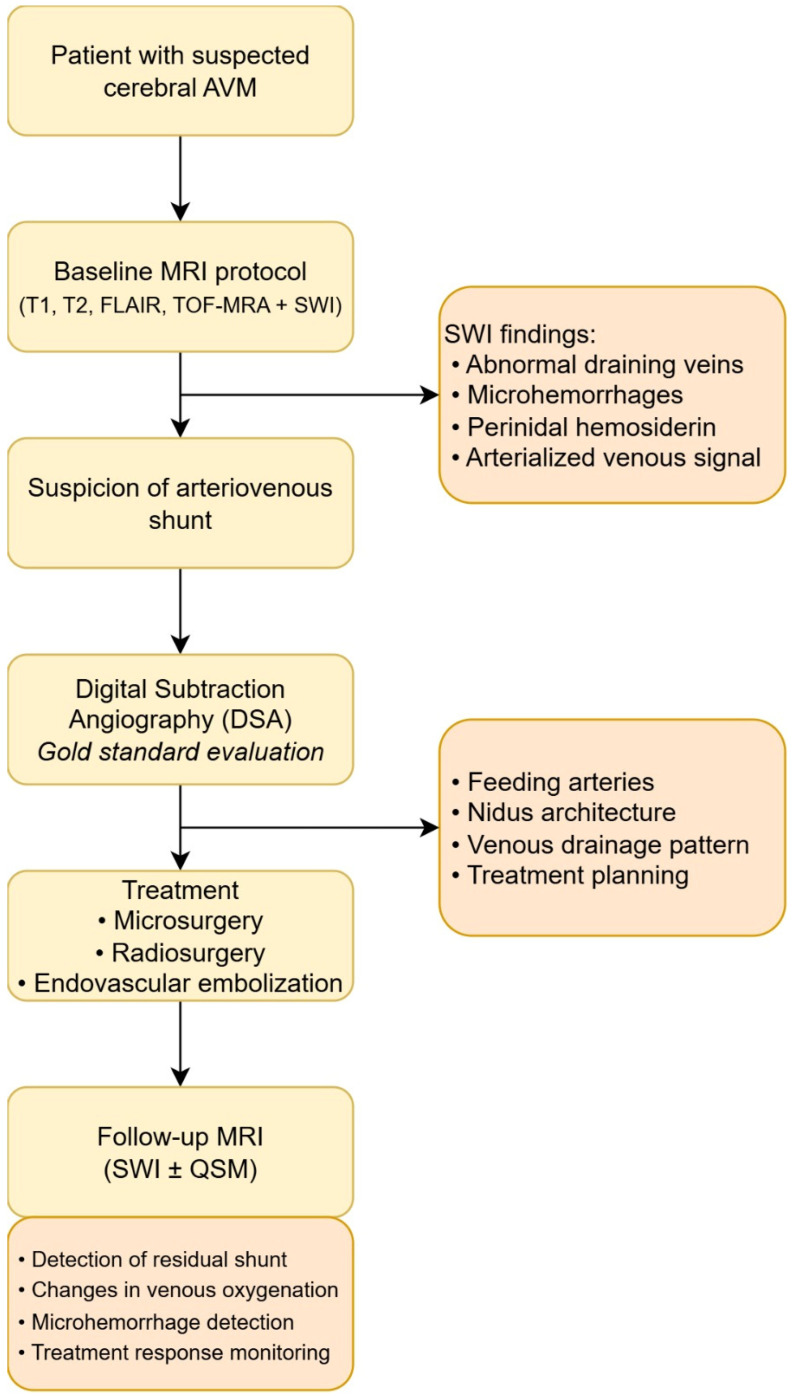
Conceptual framework linking susceptibility-based MRI findings with underlying pathophysiological mechanisms in cerebral arteriovenous malformations. Susceptibility-weighted imaging (SWI) and quantitative susceptibility mapping (QSM) provide complementary information on venous oxygenation, microhemorrhagic activity, and hemodynamic alterations, which may contribute to risk stratification and treatment monitoring.

This tiered framework preserves diagnostic rigor while progressively incorporating physiological imaging into routine AVM evaluation. Please see Figure 1, which summarizes the proposed pathophysiology-driven framework linking susceptibility-based imaging findings with AVM hemodynamics.

Baseline MRI including SWI enables non-invasive detection of abnormal venous structures, microhemorrhages, and susceptibility patterns suggestive of arterialized drainage. When an arteriovenous shunt is suspected, digital subtraction angiography (DSA) remains the reference standard for dynamic angioarchitectural characterization and treatment planning. During follow-up, susceptibility-based imaging (SWI and quantitative susceptibility mapping) may provide additional structural and physiological information regarding residual shunting, venous oxygenation changes, and treatment response.

## 11. Conclusions

Susceptibility-based magnetic resonance imaging has emerged as a valuable adjunct in the evaluation of cerebral arteriovenous malformations, bridging the gap between structural angioarchitecture and physiological insight. While digital subtraction angiography remains indispensable for dynamic vascular assessment, susceptibility-weighted imaging (SWI) and quantitative susceptibility mapping (QSM) provide complementary information that extends beyond conventional angiography.

SWI represents one of the most sensitive non-invasive tools for evaluating venous drainage in AVMs. Its high spatial resolution and intrinsic susceptibility contrast enable detailed visualization of draining veins, detection of microhemorrhages, and differentiation between hemorrhagic and calcified components. In particular, susceptibility-based depiction of arterialized veins offers insight into shunt-related hemodynamic alterations that are not directly captured by flow-based techniques.

QSM further expands the diagnostic paradigm by enabling indirect estimation of venous oxygen saturation (SvO_2_), thereby providing a quantitative window into shunt physiology. Although still investigational, QSM holds promise as a biomarker of hemodynamic modulation following radiosurgery or embolization and may contribute to refined risk stratification models in the future.

Importantly, susceptibility-based imaging does not replace DSA. Rather, it meaningfully expands preoperative and longitudinal assessment by incorporating tissue-level and physiological information. In the modern multimodal framework, SWI and QSM should be viewed as complementary tools that enhance structural delineation, biological characterization, and potentially therapeutic monitoring.

As technological refinement and prospective validation continue, susceptibility-based MRI may evolve from an adjunctive technique to an integral component of comprehensive AVM evaluation.

## Figures and Tables

**Table 1:** Comparison of imaging modalities for AVMs.

Modality	Angioarchitecture	Hemodynamics	Oxygenation	Invasiveness
DSA	Excellent	Dynamic	No	Invasive
TOF-MRA	Good arterial	Limited	No	Non-invasive
SWI	Excellent venous	Indirect	Limited	Non-invasive
QSM	Moderate	Indirect	Quantitative	Non-invasive

**Table 2:** Summary of representative studies on susceptibility-based MRI in cerebral arteriovenous malformations.

Study	Modality	Study Design	Key Findings	Limitations
George et al.	SWI	Retrospective	Improved visualization of AVM angioarchitecture	Qualitative assessment
Jagadeesan et al.	SWI	Retrospective	Detection of arteriovenous shunting	No quantitative biomarkers
Miyasaka et al.	SWI	Retrospective	Enhanced depiction of draining veins	Limited comparison with DSA
Biondetti et al.	QSM	Retrospective	Elevated venous oxygenation (SvO_2_)	Small cohort, no prospective validation
Nabavizadeh et al.	SWI/ASL	Retrospective	Improved detection of venous drainage patterns	Small sample size

Abbreviations: SWI—susceptibility-weighted imaging; QSM—quantitative susceptibility mapping; SvO_2_—venous oxygen saturation; DSA—digital subtraction angiography.

## Data Availability

No new data were created or analyzed in this study. Data sharing is not applicable.

## References

[1] Friedlander R.M. (2007). Clinical practice. Arteriovenous malformations of the brain. N. Engl. J. Med..

[2] Florian I.A., Beni L., Moisoiu V., Timis T.L., Florian I.S., Balașa A., Berindan-Neagoe I. (2021). ‘De Novo’ Brain AVMs-Hypotheses for Development and a Systematic Review of Reported Cases. Medicina.

[3] Murayama Y., Massoud T.F., Viñuela F. (1998). Hemodynamic changes in arterial feeders and draining veins during embolotherapy of arteriovenous malformations: An experimental study in a swine model. Neurosurgery.

[4] Stapf C., Mast H., Sciacca R.R., Choi J.H., Khaw A.V., Connolly E.S., Pile-Spellman J., Mohr J.P. (2006). Predictors of hemorrhage in patients with untreated brain arteriovenous malformation. Neurology.

[5] Kim H., Al-Shahi Salman R., McCulloch C.E., Stapf C., Young W.L., MARS Coinvestigators (2014). Untreated brain arteriovenous malformation: Patient-level meta-analysis of hemorrhage predictors. Neurology.

[6] McGuire L.S., Abou-Mrad T., Theiss P., Hossa J., Tshibangu M., Madapoosi A., Charbel F.T., Alaraj A. (2025). Relationship of blood flow, angioarchitecture, and rupture in cerebral arteriovenous malformations. J. Neurosurg..

[7] Hermanto Y., Takagi Y., Yoshida K., Ishii A., Kikuchi T., Funaki T., Mineharu Y., Miyamoto S. (2016). Histopathological Features of Brain Arteriovenous Malformations in Japanese Patients. Neurol. Med. Chir..

[8] Mast H., Mohr J.P., Osipov A., Pile-Spellman J., Marshall R.S., Lazar R.M., Stein B.M., Young W.L. (1995). ‘Steal’ is an unestablished mechanism for the clinical presentation of cerebral arteriovenous malformations. Stroke.

[9] Xu M., Xu H., Qin Z., Zhang J., Yang X., Xu F. (2014). Increased expression of angiogenic factors in cultured human brain arteriovenous malformation endothelial cells. Cell Biochem. Biophys..

[10] Moftakhar P., Hauptman J.S., Malkasian D., Martin N.A. (2009). Cerebral arteriovenous malformations. Part 1: Cellular and molecular biology. Neurosurg. Focus.

[11] Ajiboye N., Chalouhi N., Starke R.M., Zanaty M., Bell R. (2014). Cerebral arteriovenous malformations: Evaluation and management. Sci. World J..

[12] George U., Jolappara M., Kesavadas C., Gupta A.K. (2010). Susceptibility-weighted imaging in the evaluation of brain arteriovenous malformations. Neurol. India.

[13] Di Ieva A., Lam T., Alcaide-Leon P., Bharatha A., Montanera W., Cusimano M.D. (2015). Magnetic resonance susceptibility weighted imaging in neurosurgery: Current applications and future perspectives. J. Neurosurg..

[14] Biondetti E., Rojas-Villabona A., Sokolska M., Pizzini F.B., Jäger H.R., Thomas D.L., Shmueli K. (2019). Investigating the oxygenation of brain arteriovenous malformations using quantitative susceptibility mapping. Neuroimage.

[15] Hendee W.R., Morgan C.J. (1984). Magnetic resonance imaging. Part I—Physical principles. West. J. Med..

[16] Ogawa S., Lee T.M., Kay A.R., Tank D.W. (1990). Brain magnetic resonance imaging with contrast dependent on blood oxygenation. Proc. Natl. Acad. Sci. USA.

[17] Reichenbach J.R., Haacke E.M. (2001). High-resolution BOLD venographic imaging: A window into brain function. NMR Biomed..

[18] Logothetis N.K. (2003). The underpinnings of the BOLD functional magnetic resonance imaging signal. J. Neurosci..

[19] Wu Z., Mittal S., Kish K., Yu Y., Hu J., Haacke E.M. (2009). Identification of calcification with MRI using susceptibility-weighted imaging: A case study. J. Magn. Reason. Imaging.

[20] Haacke E.M., Xu Y., Cheng Y.C., Reichenbach J.R. (2004). Susceptibility weighted imaging (SWI). Magn. Reason. Med..

[21] Gasparotti R., Pinelli L., Liserre R. (2011). New MR sequences in daily practice: Susceptibility weighted imaging. A pictorial essay. Insights Imaging.

[22] Jagadeesan B.D., Delgado Almandoz J.E., Moran C.J., Benzinger T.L. (2011). Accuracy of susceptibility-weighted imaging for the detection of arteriovenous shunting in vascular malformations of the brain. Stroke.

[23] Miyasaka T., Taoka T., Nakagawa H., Wada T., Takayama K., Myochin K., Sakamoto M., Ochi T., Akashi T., Kichikawa K. (2012). Application of susceptibility weighted imaging (SWI) for evaluation of draining veins of arteriovenous malformation: Utility of magnitude images. Neuroradiology.

[24] Halefoglu A.M., Yousem D.M. (2018). Susceptibility weighted imaging: Clinical applications and future directions. World J. Radiol..

[25] Shmueli K., de Zwart J.A., van Gelderen P., Li T.Q., Dodd S.J., Duyn J.H. (2009). Magnetic susceptibility mapping of brain tissue in vivo using MRI phase data. Magn. Reason. Med..

[26] Wang Y., Liu T. (2015). Quantitative susceptibility mapping (QSM): Decoding MRI data for a tissue magnetic biomarker. Magn. Reason. Med..

[27] Li W., Wang N., Yu F., Han H., Cao W., Romero R., Tantiwongkosi B., Duong T.Q., Liu C. (2015). A method for estimating and removing streaking artifacts in quantitative susceptibility mapping. Neuroimage.

[28] Wharton S., Bowtell R. (2015). Effects of white matter microstructure on phase and susceptibility maps. Magn. Reason. Med..

[29] Li J., Chang S., Liu T., Wang Q., Cui D., Chen X., Jin M., Wang B., Pei M., Wisnieff C. (2012). Reducing the object orientation dependence of susceptibility effects in gradient echo MRI through quantitative susceptibility mapping. Magn. Reason. Med..

[30] Greenberg S.M., Vernooij M.W., Cordonnier C., Viswanathan A., Al-Shahi Salman R., Warach S., Launer L.J., Van Buchem M.A., Breteler M.M. (2009). Microbleed Study Group. Cerebral microbleeds: A guide to detection and interpretation. Lancet Neurol..

[31] Haacke E.M., Mittal S., Wu Z., Neelavalli J., Cheng Y.C. (2009). Susceptibility-weighted imaging: Technical aspects and clinical applications, part 1. AJNR Am. J. Neuroradiol..

[32] Wilms G., Bosmans H., Demaerel P., Marchal G. (2001). Magnetic resonance angiography of the intracranial vessels. Eur. J. Radiol..

[33] Nabavizadeh S.A., Edgar J.C., Vossough A. (2014). Utility of susceptibility-weighted imaging and arterial spin perfusion imaging in pediatric brain arteriovenous shunting. Neuroradiology.

[34] Yamaguchi S., Hamabe J., Horie N., Iki Y., Sadakata E., Hiu T., Yagi N., Suyama K. (2020). Hypointensity of draining veins on susceptibility-weighted magnetic resonance images might indicate normal venous flow and a lower risk of intracerebral hemorrhage in patients with intracranial arteriovenous shunt(s). J. Clin. Neurosci..

[35] Byun J., Kwon D.H., Lee D.H., Park W., Park J.C., Ahn J.S. (2020). Radiosurgery for Cerebral Arteriovenous Malformation (AVM): Current Treatment Strategy and Radiosurgical Technique for Large Cerebral AVM. J. Korean Neurosurg. Soc..

[36] Jain R., Robertson P.L., Gandhi D., Gujar S.K., Muraszko K.M., Gebarski S. (2005). Radiation-induced cavernomas of the brain. AJNR Am. J. Neuroradiol..

[37] Shaban S., Huasen B., Haridas A., Killingsworth M., Worthington J., Jabbour P., Bhaskar S.M.M. (2022). Digital subtraction angiography in cerebrovascular disease: Current practice and perspectives on diagnosis, acute treatment and prognosis. Acta Neurol. Belg..

[38] Willinsky R.A., Taylor S.M., TerBrugge K., Farb R.I., Tomlinson G., Montanera W. (2003). Neurologic complications of cerebral angiography: Prospective analysis of 2,899 procedures and review of the literature. Radiology.

[39] Oleaga L., Dalal S.S., Weigele J.B., Hurst R.W., Lee J., Voorhees A., Melhem E.R. (2010). The role of time-resolved 3D contrast-enhanced MR angiography in the assessment and grading of cerebral arteriovenous malformations. Eur. J. Radiol..

[40] Weber W., Kis B., Siekmann R., Kuehne D. (2007). Endovascular treatment of intracranial arteriovenous malformations with onyx: Technical aspects. AJNR Am. J. Neuroradiol..

[41] Deistung A., Schäfer A., Schweser F., Biedermann U., Turner R., Reichenbach J.R. (2013). Toward in vivo histology: A comparison of quantitative susceptibility mapping (QSM) with magnitude-, phase-, and R_2_*-imaging at ultra-high magnetic field strength. Neuroimage.

[42] Salman F., Ramesh A., Jochmann T., Prayer M., Adegbemigun A., Reeves J.A., Wilding G.E., Cho J., Jakimovski D., Bergsland N. (2025). Sensitivity of Quantitative Susceptibility Mapping for Clinical Research in Deep Gray Matter. Hum. Brain Mapp..

[43] Wei H., Cao S., Zhang Y., Guan X., Yan F., Yeom K.W., Liu C. (2019). Learning-based single-step quantitative susceptibility mapping reconstruction without brain extraction. Neuroimage.

[44] Sun Y., Guo H., Li M., Zhang Z. (2026). Nonlinear inversion model-driven deep learning method for magnetic resonance imaging (MRI) quantitative susceptibility mapping imaging. Quant. Imaging Med. Surg..

[45] Basilio-Flores J.E., Aguilar-Melgar J.A., Pacheco-Fernandez Baca H. (2025). Predicting the natural history of unruptured brain arteriovenous malformations: External validation of rupture risk scores. J. Neurosurg..

[46] Hanspach J., Bollmann S., Grigo J., Karius A., Uder M., Laun F.B. (2022). Deep learning-based quantitative susceptibility mapping (QSM) in the presence of fat using synthetically generated multi-echo phase training data. Magn. Reason. Med..

[47] Si W., Guo Y., Zhang Q., Zhang J., Wang Y., Feng Y. (2023). Quantitative susceptibility mapping using multi-channel convolutional neural networks with dipole-adaptive multi-frequency inputs. Front. Neurosci..

